# Incorporating a new criteria of resectability for pancreatic ductal adenocarcinoma

**DOI:** 10.1590/0102-67202025000017e1886

**Published:** 2025-07-21

**Authors:** Marcos BELOTTO, Luís Felipe Leite DA SILVA, Renata D’Alpino PEIXOTO, Eduardo de Souza Martins FERNANDES, Orlando Jorge Martins TORRES

**Affiliations:** 1 Hospital 9 de Julho, Department of Surgery – São Paulo (SP), Brazil.; 2 Universidade Federal Fluminense, Medical Science Department – Rio de Janeiro (RJ), Brazil.; 3 University of British Columbia, Medical Oncology Department – Vancouver, Canada.; 4 Hospital São Lucas, Department of Hepatopancreatobiliary and Transplant – Rio de Janeiro (RJ), Brazil.; 5 Universidade Federal do Maranhão, Hospital Universitário Presidente Dutra, Department of Hepatopancreatobiliary and Liver Transplant – São Luis (MA), Brazil.

**Keywords:** Pancreatic neoplasms, Pancreatectomy, Pancreaticoduodenectomy, Survival, Neoplasias pancreáticas, Pancreatectomia, Pancreaticoduodenectomia, Sobrevida

## Abstract

The classification of resectability for pancreatic ductal adenocarcinoma is crucial in guiding treatment strategies. A recent system including anatomic (A), biological (B), and conditional (C) factors has been used to select the patients who underwent pancreatoduodenectomy, and centralization has been associated with low mortality and defined as a critical determinant of surgical outcomes. A comprehensive literature review assessed the impact of incorporating the ABC criteria in patients with pancreatic ductal adenocarcinoma. Incorporating biological and conditional criteria for patients with pancreatic ductal adenocarcinoma could enhance patient stratification accuracy and improve clinical outcomes and survival.

## ARTICLE HIGHLIGHTS

- The classification of resectability in pancreatic ductal adenocarcinoma has evolved from purely anatomic criteria to include biological and conditional factors, improving patient selection for surgery
- The ABC (Anatomic, Biological, Conditional) system proposed by the International Association of Pancreatology refines preoperative assessment and guides the decision between initial resection or neoadjuvant therapy.
- Elevated serum CA 19–9 levels above 500 U/mL, even in anatomically resectable tumors, are associated with worse survival and may indicate the need for neoadjuvant chemotherapy.
- he MetroPancreas model, based on tumor size and CA 19–9 levels, is a useful tool for predicting the futility of pancreatectomy and supporting individualized clinical decision-making.

## CENTRAL MESSAGE

Locally advanced pancreatic ductal adenocarcinoma is typically considered unresectable due to major vascular involvement. In recent years, the treatment paradigm for borderline resectable and locally advanced has shifted significantly, with neoadjuvant therapy emerging as the standard of care. This approach, often preferred over upfront surgery, aims to downstage the tumor, reduce the risk of positive margins, and increase the probability of a successful resection and survival.

## PERSPECTIVES

Anatomical, biological, and conditional factors should be incorporated into clinical practice for the preoperative staging of patients with pancreatic ductal adenocarcinoma. These factors are essential for deciding whether to perform upfront resection or neoadjuvant chemotherapy and optimize outcomes.

## VISUAL ABSTRACT

**Figure:**
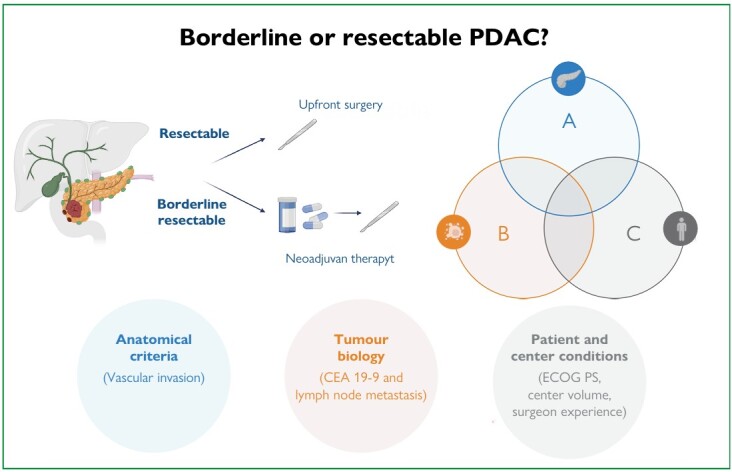

## RESUMO

A classificação da ressecabilidade no adenocarcinoma ductal pancreático é fundamental para orientar as estratégias terapêuticas. Um sistema recente, que incorpora fatores anatômicos (A), biológicos (B) e condicionais (C), tem sido utilizado na seleção de pacientes submetidos à pancreatoduodenectomia, sendo que a centralização do tratamento tem se associado à baixa mortalidade e se consolidado como determinante crítico dos desfechos cirúrgicos. Esta revisão abrangente da literatura avaliou o impacto da incorporação dos critérios ABC em pacientes com adenocarcinoma ductal pancreático. A inclusão de critérios biológicos e condicionais pode aprimorar a estratificação dos pacientes, melhorando os desfechos clínicos e a sobrevida.

## INTRODUCTION

 Pancreatic ductal adenocarcinoma (PDAC) presents a dismal overall prognosis and is a major cause of cancer-related mortality worldwide. Whereas less than 20% of PDAC patients have resectable disease, most patients present with locally advanced or metastatic disease. Achieving a negative-margin (R0) resection is widely recognized as a crucial factor in prolonging survival in PDAC patients and reducing locoregional recurrence. However, for many surgeons, this goal remains elusive due to the tumor’s proximity to vital vascular structures, making surgical intervention highly complex^4,5,15,20,24^ . 

 Borderline resectable PDAC (BR-PDAC) and locally advanced PDAC (LA-PDAC) represent distinct and challenging categories between clearly resectable and unresectable diseases^8, 21^. BR-PDAC was introduced to identify tumors with limited vascular involvement, where the likelihood of R0 resection is uncertain but achievable with appropriate treatment strategies^9^ . Locally advanced pancreatic ductal adenocarcinoma (LA-PDAC) is defined as a tumoral condition considered unresectable due to vascular involvement. In recent years, the treatment paradigm for BR-PDAC and LA-PDAC has shifted significantly, with neoadjuvant therapy emerging as the standard of care^10,23^. This approach, often preferred over upfront surgery, aims to downstage the tumor, reduce the risk of positive margins, and increase the probability of a successful resection^1,2,4,12,14-17^. Concurrently, advancements in surgical techniques, such as the artery first approach, vascular resection and reconstruction, arterial divestment, and total mesopancreas excision, have pushed the boundaries of what is considered resectable and improved R0 rates, offering new hope for patients with previously inoperable tumors^3,7 ,9,10,19^. 

 The International Association of Pancreatology (IAP) in 2017 proposed to expand the preoperative staging criteria by redefining borderline resectable PDAC with biological and conditional criteria, suggesting that resectability status should be assessed beyond the anatomic relationship between tumor and vessels. The IAP definition represents a significant advancement in the assessment and management of PDAC, aiming to improve the accuracy of resectability predictions and better guide treatment decisions^13^ . The anatomical criteria define a tumor that is at high risk for margin-positive resection when upfront surgery is used as an initial treatment strategy. The biological criteria are defined when there are findings that raise the possibility of extra-pancreatic metastatic disease. The conditional criteria are defined when the patient has a high risk for mortality or complications after surgery related to performance status and co-morbidities^13^ . 

 The evolution of PDAC classification has mirrored these advancements, from focusing solely on anatomical criteria to including disease biology and patient-specific factors such as performance status and comorbidities^14^ . The size and location of the tumor represent anatomical factors. Biological (biochemical) factors are represented by elevated preoperative serum carbohydrate antigen (CA 19–9) above 500 IU/ mL and biopsy-proven regional lymph node metastasis, which were associated with more aggressive tumor behavior and a higher risk of occult metastasis. This allows for the identification of patients who may be better candidates for neoadjuvant therapy, thereby improving disease recurrence rates. Equally important are the conditional criteria, represented by the Eastern Cooperative Oncology Group (ECOG) performance status, which includes elements that significantly influence surgical outcomes. A score of two or higher serves as a criterion for BR-PDAC under the conditional category^24^ . This reflects the increased risk of postoperative complications and poorer overall prognosis in patients with compromised physiological reserves. The inclusion of conditional factors ensures that treatment strategies are tailored not only to the tumor’s characteristics but also to the patient’s ability to tolerate and benefit from aggressive resection, thereby optimizing the balance between radicality and surgical safety^6,8 ,11,20^ . 

 Incorporating biological and conditional criteria is an important tool for patients with PDAC to guide better patient stratification during the treatment and improve survival outcomes. High-volume centers with specialized multidisciplinary teams have adopted this system and observed that this system is consistently associated with improved quality of decision-making and offers more patients surgical treatment^6,7,11,20^ . 

 In the previous classification of PDAC based on anatomical parameters, many systems have been used to classify the extent of PDAC (NCCN, Americas Hepato-Pancreato-Biliary Association [AHPBA]/Society of Surgical Oncology [SSO]/ Society of Surgical Alimentary Tract [SSAT], MD Anderson Cancer Center (MDACC), Alliance)^1, 2,4,5 ,13^. These classifications have been based on the tumor’s relationship with surrounding vascular structures to determine resectability, and the most common is the National Comprehensive Cancer Network’s (NCCN) classification (Table 1)^22^ . 

**Table 1:** Radiological criteria to classify resectability based on anatomical parameters (NCCN)^22^ .

Resectable
Superior mesenteric vein/portal vein
No tumor contact or ≤180° contact without vein contour irregularity
Superior mesenteric artery
No solid tumor contact
Common hepatic artery and its first-order branches
No solid tumor contact
Coeliac trunk
No solid tumor contact
Borderline resectable
Superior mesenteric vein/portal vein
Solid tumor contact measuring >180°, or solid tumor contact ≤180° with contour irregularity or thrombosis
Superior mesenteric artery
Solid tumor contact ≤180°
Common hepatic artery and its first-order branches
Solid tumor contact without extension to the coeliac artery or hepatic artery bifurcation
Coeliac trunk
Solid tumor contact ≤180°
Locally advanced
Superior mesenteric vein/portal vein
Unreconstructable
Superior mesenteric artery
Solid tumor contact >180°
Common hepatic artery and its first-order branches
Unreconstructable
Coeliac trunk
Solid tumor contact >180°

 The literature provided further refinement, maintaining a strong anatomical focus while introducing subclassifications based on vascular involvement. Resectable tumors were those with minimal or no vascular involvement, while BR-PDAC was divided into BR-PV (venous invasion alone) and BR-A (arterial invasion)^1,2,4,5^. Some systems uniquely emphasized the importance of both the degree of vascular involvement and the specific vessels affected. For instance, BR-PDAC with both venous and arterial involvement was graded as BR-A, given the worse prognosis and higher risk of incomplete resection associated with arterial involvement. Additionally, the classification considered the tumor’s extension relative to the inferior border of the duodenum, with involvement beyond this point considered unresectable^14-18^. 

 Crippa et al.^6^ developed a futility risk model for patients undergoing upfront pancreatectomy. The concept of high-risk patients is mainly qualitative, as the definition includes large primary tumors, elevated CA 19–9 serum levels, nodal metastases on imaging, significant pain, and excessive weight loss. It is important to analyze the pretreatment variables associated with futile resection, which include death from postoperative complications, disease recurrence within 6 months, and PDAC-related events (very early recurrence). The MetroPancreas is an online calculator designed to quantify the likelihood of futility of a specific patient and might be useful in selecting patients for upfront resection or neoadjuvant chemotherapy^12^ . 

 The concept identified four discrete conditions defined as CA 19–9 adjusted to tumor size to keep the likelihood of futile pancreatectomy below 20% (0.80)^6,12^.

- size <2 cm with CA 19–9 <1000 U/mL,
- size <3 cm with CA 19–9 <500 U/mL,
- size <4 cm with CA 19–9 <150 U/mL
- size <5 cm with CA 19–9 <50 U/mL

Schouten et al. evaluated the prognostic value of anatomical, biological, and conditional factors for staging patients with resectable PDAC. This study observed that survival after upfront resection in anatomically resectable patients is significantly worse if the biological factor is unfavorable for PDAC (serum CA 19–9 ≥500 U/mL). The ECOG performance status did not impact survival in this group of patients. These findings suggest that serum CA 19–9 levels are valuable for preoperative staging of patients with resectable PDAC to decide whether to undergo upfront resection or neoadjuvant chemo (radio) therapy^20^. 

## CONCLUSIONS

Anatomical, biological, and conditional factors should be incorporated into clinical practice for the preoperative staging of patients with PDAC. These factors are essential for deciding whether to perform upfront resection or neoadjuvant chemotherapy and optimize outcomes and survival.

## Data Availability

The informations regarding the investigation, methodology and data analysis of the article are archived under the responsibility of the authors.
